# Overexpression of aldo-keto-reductase in azole-resistant clinical isolates of *Candida glabrata* determined by cDNA-AFLP

**DOI:** 10.1186/2008-2231-21-1

**Published:** 2013-01-02

**Authors:** Shirin Farahyar, Farideh Zaini, Parivash Kordbacheh, Sassan Rezaie, Mahin Safara, Reza Raoofian, Mansour Heidari

**Affiliations:** 1 Department of Medical Mycology and Parasitology, School of Public Health, Tehran University of Medical Sciences, Tehran, Iran; 2 Department of Medical Genetics, School of Medicine, Tehran University of Medical Sciences, Tehran, Iran; 3 Department of Medical Genetics, School of Medicine, Mashhad University of Medical Sciences, Mashhad, Iran

**Keywords:** Azole, Aldo-keto-reductase, cDNA-AFLP, *Candida glabrata*, Semi-quantitative RT-PCR

## Abstract

**Background:**

*Candida glabrata* causes significant medical problems in immunocompromised patients. Many strains of this yeast are intrinsically resistant to azole antifungal agents, and treatment is problematic, leading to high morbidity and mortality rates in immunosuppressed individuals. The primary goal of this study was to investigate the genes involved in the drug resistance of clinical isolates of *C. glabrata.*

**Methods:**

The clinical isolates of *C. glabrata* were collected in an epidemiological survey of candidal infection in immunocompromised patients and consisted of four fluconazole and itraconazole resistant isolates, two fluconazole and itraconazole sensitive isolates, and *C. glabrata* CBS 138 as reference strain. Antifungal susceptibility patterns of the organisms were determined beforehand by the Clinical and Laboratory Standards Institute (CLSI). The potential gene(s) implicated in antifungal resistance were investigated using complementary DNA- Amplified Fragment Length Polymorphism (cDNA-AFLP). Semi-quantitative RT-PCR was carried out to evaluate the expression of gene(s) in resistant isolates as compared to sensitive and reference strains.

**Results and conclusions:**

The aldo-keto-reductase superfamily (*AKR* gene) was upregulated in the resistant clinical isolates as assessed by cDNA-AFLP. Semi-quantitative RT-PCR revealed AKR mRNA expression approximately twice that seen in the sensitive isolates. Overexpression of the *AKR* gene was associated with increased fluconazole and itraconazole resistance in *C. glabrata*. The data suggest that upregulation of the *AKR* gene might give a new insight into the mechanism of azole resistance.

## Background

The incidence of fungal infections has increased over the past two decades. Opportunistic fungal infections occur in immunocompromised hosts [1], particularly among patients infected with human immunodeficiency virus (HIV), individuals receiving immunosuppressive therapy for organ or stem cell transplantation, and cancer patients [2]. *Candida glabrata* has emerged as a common fungal pathogen in many countries and is often reported as the second most prevalent species after *C. albicans*[3]. *Candida glabrata* appears to be innately resistant to fluconazole [4,5] and is less sensitive to it than *C. albicans*, *C. parapsilosis*, or *C. tropicalis.* Studies have shown that mechanisms of azole resistance in *C. glabrata* are often associated with the upregulation of the genes *CgCDR1* (*C. glabrata Candida* drug resistance), *CgCDR2* (formerly *PDH1*) [6,7], and *CgSNQ2* (*C. glabrata* sensitivity to 4-nitroquinoline N-oxide) which encode proteins belonging to ATP binding cassette (ABC) transporters [8]. *CgSNQ2* was controlled by *CgPDR1* (*C. glabrata* pleiotropic drug resistance 1) [9]. A further azole resistance mechanism in *C. glabrata* has been shown to be the mutation or overexpression of the *ERG11* gene (*CgERG11*) that encodes cytochrome P-450 lanostrol 14-α demethylase, the azole target enzyme, and increases *CYP51* (the *ERG11* orthologue) activity, enhancing azole resistance [10]. *ERG11* is a well-characterized gene implicated in the fluconazole resistance of *Candida krusei* and *Candida albicans*[6,11,12]. The molecular mechanisms in the resistant strains of *C. glabrata* are not completely understood. In this study, antifungal resistance mechanisms were investigated using cDNA-AFLP, a genome-wide expression analysis method that does not require prior sequence knowledge of the genes [13]. This technology is an ideal alternative to microarrays [14].

## Materials and methods

### Yeast isolates

Four fluconazole and itraconazole resistant isolates, two fluconazole and itraconazole sensitive isolates, and one reference strain of *C. glabrata* were used. The isolates of *C. glabrata* were obtained from clinical samples collected in an epidemiological survey of candidal infections in immunocompromised patients conducted at the Department of Medical Mycology and Parasitology, Tehran. The clinical isolates were recovered from oropharynx of patients. The reference *C. glabrata* strain, CBS 138, was provided by Professor Koichi Makimura, Teikyo University Institute of Medical Mycology-Tokyo, Japan (Table 1). The clinical isolates were identified using standard mycological methods, including assimilation patterns.

**Table 1:** **Number of isolates, site of isolation and azole susceptibility of *Candida glabrata *clinical isolates used in this study**

**Isolate no.**	**Site of isolation**	**MIC(μg/ml)**	
		**Fluconazole**	**Itraconazole**
94	Oropharynx	0.25	0.5
45	Oropharynx	0.5	0.25
51	Oropharynx	64	2
153	Oropharynx	64	4
137	Oropharynx	64	2
219	Oropharynx	64	2

### Culture conditions and drug susceptibility testing

The isolates and reference strain were cultured on a yeast extract peptone-glucose (YEPD) agar plate containing 5 g/l yeast extract (Baltimore Biological Laboratory, USA), 10 g/l peptone (Merck, Germany), 20 g/l glucose (Merck, Germany), 0.5 g/l chloramphenicol; and 20 g/l agar (Biolife, Italy) incubated at 37°C for 72 h. A single colony of each isolates and reference strain was selected and subcultured in YEPD broth for 24 h at 37°C. Resistant isolate No. 51 was cultured in YEPD broth with fluconazole (128 μg/ml) for 48 h at 37°C. Susceptibility to fluconazole and itraconazole drugs was determined by microdilution according to the criteria for Clinical and Laboratory Standards Institute (CLSI) M27–A2 (formerly NCCLS) [15,16].

### RNA extraction

Total RNA was extracted from the exponential phase using the RNeasy Protect Mini Kit (Qiagen, Germany). For mechanical disruption, the yeast cells were sonicated with acid-washed glass beads (0.45–0.52 mm diameter). The released RNA was treated through an RNase-free DNase step (Qiagen, Germany) and quantity and quality of RNA was measured with the Nanodrop 1000 spectrophotometer (Thermo Scientific, USA).

### cDNA-AFLP

Complementary DNA Amplified Fragment Length Polymorphism (cDNA-AFLP) was conducted with minor modifications. For complementary DNA (cDNA) synthesis, 6 μg of total RNA from each isolate was heated at 65°C for 10 min, followed by cooling on ice. A master mixture contained 7.5 μl of 5X reverse transcriptase (RT) buffer comprising Tris–HCl (pH 8.3 at 25°C) (Fermentas, Canada); 1 μl Oligo dT (20 pmol/μl); all four dNTPs (10 mM) 1.5 μl; Ribolock (20 U) 1.5 μl (Fermentas, Canada); and DEPC treated water. Two hundred units of Moloney Murine Leukemia Virus (M-MuLV) reverse transcriptase enzyme (Fermentas) were added. The RT temperature was 42°C for 60 min and 70°C for 10 min. cDNA was checked with the reference gene *URA3* (orotidine-5’-phosphate decarboxylase) with the following PCR conditions: 5 min at 94°C; 30 cycles of 30 s at 94°C, 30 s at 55°C, 45 s at 72 °C, and 7 min at 72°C. Primers were designed with the NCBI primer–BLAST (Basic Local Alignment Search Tool) program http://www.ncbi.nih.gov/primer-BLAST (Table 2). DNA polymerase I (Fermentas, Canada) was used for second strand cDNA synthesis at 16°C for 3 h and was precipitated with ethanol. The quality of dscDNA was evaluated with the Nanodrop 1000 spectrophotometer (Thermo Scientific). Two micrograms dscDNA were digested with the MboI restriction enzyme (Fermentas) for 4 h at 37°C, and the enzyme was inactivated at 80°C for 20 min. Eight μg of ADMbo1 and 4 μg of adMbo1 cDNA-AFLP adaptors (Table 3) were ligated to MboI digested dscDNA fragments by T4 DNA Ligase (Takara Bio Inc. Japan) conducted at 1 min at 50°C, decreasing to 10°C over the course of 1 h (1°C per 90 s). T4 DNA ligase was added, and the mixture was incubated at 16°C for 16 h. The pre-amplification was performed with the PreAmp adaptor as primer with the following PCR conditions: 5 min of denaturation at 94°C; 30 cycles of 94°C for 30 s, 55°C for 30 s, 72°C for 30 s; and a final extension at 72°C for 5 min. The sensitive adaptors used in sensitive amplification were designed with one selective base at the 3′end (Table 3). Ten PCRs were performed utilizing all sensitive adaptor combinations, and the PCR products were separated by 8% non-denaturated poly acrylamide gel electrophoresis (PAGE) and stained with silver nitrate. The differentiated transcription-derived fragments (TDFs) were observed.

**Table 2:** Primers used in this study

**Gene**	**Primer**	**Sequence**	**Gene location (5′-3′)**	**Product size (bp)**
URA3	URA3 F	GGGCTCTTTAGCTCATGGTG	432-451	173
	URA3 R	CAAGTGCATCGCCTTTATCA	604-585	
AKR	AKR F	GGTCTCGGGCCTCGGCTACA	321-340	289
	AKR R	TGGGGCATACGCCTCGACCA	609-590	

**Table 3:** Adaptors used in cDNA-AFLP method

**Adaptors**	**Sequence (5′-3′)**
ADMbo1	AGCACTCTCCAGCCTCTCACCGCA
adMbo1	GATCTGCGGTGA
Pre Amp	AGCACTCTCCAGCCTCTCACCGCAGATC
S1Mbo1	AGCACTCTCCAGCCTCTCACCGCAGATCC
S2Mbo1	AGCACTCTCCAGCCTCTCACCGCAGATCG
S3Mbo1	AGCACTCTCCAGCCTCTCACCGCAGATCA
S4Mbo1	AGCACTCTCCAGCCTCTCACCGCAGATCT

### Isolation, cloning, and sequencing of cDNA-AFLP fragments

Selected bands were isolated from the PAGE and re-amplified using appropriate sensitive adaptors. The TDFs were cloned using a TA-cloning kit (Invitrogen, USA) and the recombinant plasmids were screened using M13 forward (−20) (5′-GTAAAACGACGGCCAG-3′) and M13 reverse (5′-CAGGAAACAGCTATGAC-3′) primers with the following PCR protocol: 30 cycles of 94°C for 1 min, 55°C for 1 min; 72 C for 1 min; and 72°C for 7 min. PCR products were analyzed by agarose gel electrophoresis . The recombinant plasmids containing unknown DNA were sequenced by M13 forward (−20) and M13 reverse primers. Some TDFs were verified with direct sequencing (Macrogene, Korea). Sequence data were analyzed in non-redundant nucleic and protein databases BLAST (http://www.ncbi.nim.nih.gov/BLAST/).

### Semi-quantitative RT-PCR

Semi-quantitative RT-PCR was carried out to evaluate the mRNA overexpression level of TDFs, which were identified by cDNA-AFLP [17] using specifically designed primers (Table 2). An equal amount (6 μg) of total RNA from each of the clinical isolates and the CBS 138 reference strain was used for first strand cDNA synthesis, and the expression pattern in cDNA-AFLP was determined with the primers on RNA of clinical isolates. The *URA3* gene was used as internal control and negative controls were prepared with sterile water as template. The gel image was captured digitally with a Sony XC-ST50CE camera (Sony, Japan). The band intensity was analyzed and quantified with gel analysis software UVI (Roche, Germany).

## Results

### Antifungal susceptibility

Susceptibility to fluconazole and itraconazole was determined by the broth microdilution method described in the CLSI (document M27-A2). The minimum inhibitory concentration (MIC) of fluconazole and itraconazole obtained against clinical isolates of *C. glabrata* showed four strains resistant to fluconazole (MIC 64 μg/ml) and itraconazole (MIC 2–4 μg/ml), while two strains were susceptible to fluconazole (MIC 0.25–0.50 μg/ml) and itraconazole (MIC 0.25–0.50 μg/ml) (Table 1).

### cDNA-AFLP

#### The cDNA-AFLP products of clinical isolates and reference strain

**Figure 1:**
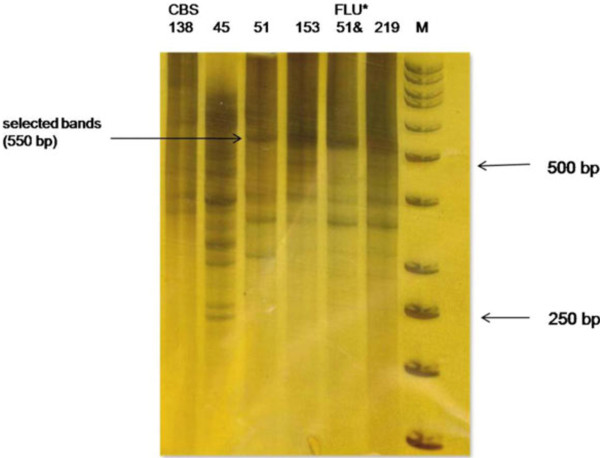
**Expression pattern of TDFs of cDNA-AFLP PAGE.** Sensitive amplification of cDNA-AFLP on a PAGE from S3Mbo1/S4Mbo1 as primers. The lanes numbered correspond to the clinical isolates presented in Table 1. The arrows point to a differentially expressed TDFs. M: Marker 50 bp; * FLU: Fluconazole.

**Figure 2:**
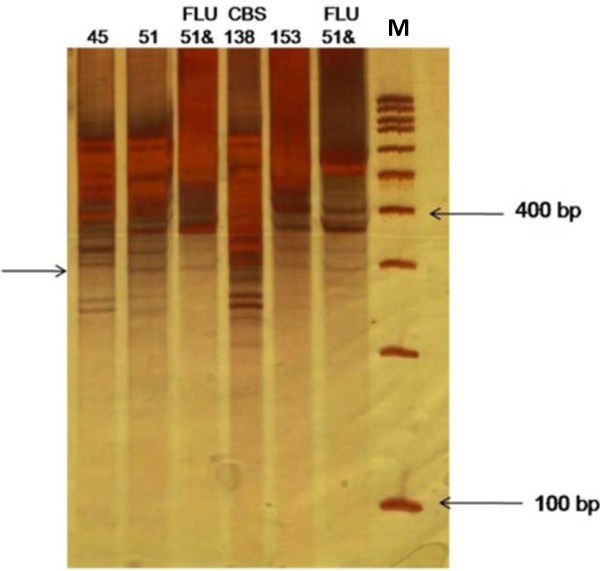
**Expression pattern of TDFs of cDNA-AFLP PAGE using silver staining.** Sensitive amplification of cDNA-AFLP on a PAGE from primer combinations S2Mbo1/S2Mbo1 and S2Mbo1/S3Mbo1. The lanes numbered correspond to the clinical isolates presented in Table 1. The arrows point to differentially expressed TDFs. DNA ladder; M: (100 bp) molecular weight marker. *FLU: Fluconazole.

Fragments of cDNA-AFLP were observed on 8% non-denaturing PAGE using silver staining. Over 100 TDFs were produced using 10 primer combinations. Twenty fragments were identified as differentially regulated. High expression TDFs were observed in the resistant isolates, with lower levels of cDNA-AFLP fragments detected in the sensitive isolates. Ten TDFs ranging from 120 bp to 600 bp were isolated from the cDNA-AFLP profile and identified by cloning and DNA sequencing (Table 4). A differentially expressed TDF was produced at approximately 550 bp when using S3Mbo1 and S4Mbo1 as sensitive primers (Figure 1). Sequencing showed that the TDF was consistent with the gene associated with aldo-keto-reductase (*AKR* gene: Gene ID: 2886902; Gene Symbol: CAGL0C04543g; XM_445372.1). Other clones contained two unknown function sequences observed at about 320 bp and 360 bp using S2Mbo1/S2Mbo1 and S2Mbo1/S3Mbo1 as sensitive primers, respectively (Figure 2).

**Table 4:** Sequences identified by cDNA-AFLP

**Accession no.**	**Size (bp)**	**Identity**
XM_445372.1	550	aldo-keto-reductase
XM_446417.1	320	unknown function
XM_444814.1	360	unknown function

### Semi-quantitative RT-PCR

**Figure 3:**
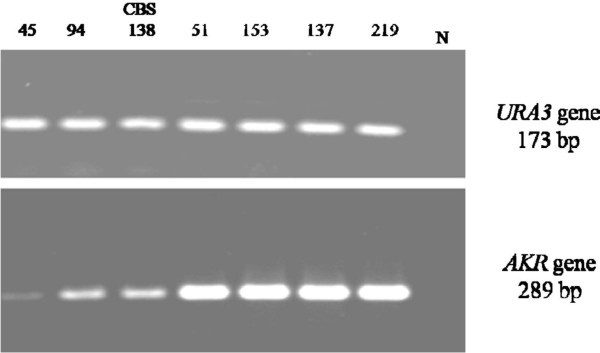
**Semi quantitative RT-PCR analysis of *AKR *gene mRNA expression.** The lanes numbered correspond to the clinical isolates presented in Table 1. N, negative control (water). *URA3* gene (173 bp) was used as internal control.

**Figure 4:**
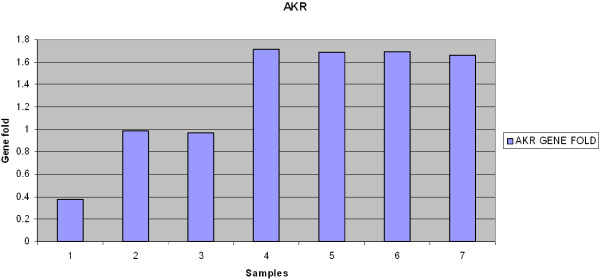
***AKR *gene showed increased expression level after normalization with internal control (*URA3*).** Samples 1 and 2 show clinical isolates of *C*. *glabrata* sensitive to fluconazole and itraconazole; 3 shows CBS 138, and samples 4,5,6,7 are resistant clinical isolates.

To confirm AKR as a potential gene for azole resistance, a semi-quantitative RT-PCR technique was performed on the cDNAs from resistant and sensitive clinical isolates as well as on the CBS 138 reference strain. Figure 3 represents the upregulation of AKR mRNA expression levels in these samples. Semi-quantitative RT-PCR showed that AKR mRNA expression was about twice that seen in the sensitive isolates (Figure 4). AKR transcript level in the resistant isolate treated with fluconazole was the same as the one observed in the resistant clinical isolates (data not shown).

## Discussion

*Candida glabrata*, second only to *C. albicans* as an infectious pathogen in candidiasis, contributes to an average of 11% of candidal infections, varying from 7% to 20% depending on geographic location [18]. In the survey providing data for the present study of immunocompromised patients, *C. glabrata* accounted for about 15% of candidal infections. Twenty-five percent of the isolates were resistant to fluconazole (Zaini *et al.*, unpublished data). Although several genes may be implicated in azole resistance, the molecular pathways involved are not completely understood.

We report, for the first time using cDNA-AFLP, AKR transcripts upregulated in resistant clinical isolates. The *AKR* gene is not the first suggested to be involved in azole resistance. Upregulation of *CgCDR1* and *CgCDR2* genes associated with increased expression of the ABC transporter has been well documented [19,20]. The *PDR1* gene is important in acquired azole resistance [21], and so-called gain-of-function mutations of the *CgPDR1* gene have been shown to play an essential role in azole resistance by *C. glabrata*[22-25]. These mutations indicate that many genes are differentially regulated in azole resistant isolates as compared to the wild type. Multiple genes (from 27–235) show increased expression, and aldo-keto-reductase (CAGL0C04543g: XM_445372.1) is upregulated (1.5 to 2-fold) [25]. The results of the present study also demonstrated that *AKR* mRNA expression in azole resistance reaches levels of about twice that found in sensitive strains. The molecular mechanisms of azole resistance in *C. albicans* are connected with overexpression of ATP-binding cassette (ABC) transporters or major facilitator superfamily, and upregulation of these genes can also be brought about by exposure to benomyl and fluphenazine. In the presence of benomyl, some genes belonging to the aldo-keto reductase family, such as *IFD* genes*, IPF5987*, and *GRP2*, can be activated with the oxido-reductase function [26].

The aldo-keto-reductase superfamily (AKR) comprises several proteins with similar kinetic and structural properties and has been found in a wide range of phyla, including both prokaryotes and eukaryotes [27]. AKRs catalyze the reduction of aldehydes and ketones to their corresponding alcohol products by reducing nicotinamide adenine dinucleotide phosphate (NADPH) cofactor [28]. Several drugs and pharmaceuticals are reactive carbonyls and aldehydes or are converted to carbonyls during *in vivo* metabolism. An important role of AKRs is in preventing carbonyl toxicity [27]. The physiological roles of this superfamily have been studied in the yeast *Saccharomyces cerevisiae*, a simple eukaryote containing various *AKR* genes that encode proteins similar in structure and function to mammalian AKRs, including those of humans. The physiological activity of yeast AKRs is largely unclear. Prior studies have identified six open reading frames (YHR104W, YOR120W, YDR368W, YBR149W, YJR096W, YDL124W) in the *S*. *cerevisiae* genome that encode proteins with activity overlapping human aldose reductase [29]. YHR104W and YDR368W are stress response proteins [30]. The product of YOR120W is a galactose-inducible crystalline-like yeast protein, and YBR149W encodes a dehydrogenase that plays a role in the direction of arabinose oxidation rather than reduction [31]. Numerous studies have demonstrated that aldo-keto-reductases are active in stress conditions. Another important role of the yeast *AKR* genes is to protect against heat shock stress [32]. In addition, AKRs potentially play roles in oxidative defense and transcriptional regulation [33]. AKR function has also been reported in drug metabolism and detoxification of pharmaceuticals, drugs, and xenobiotics in humans [27], and it seems that the physiological roles of the *AKR* gene in yeast are similar to those in humans. Our results suggest upregulation of *AKR* gene is involved in the molecular mechanism of drug resistance in *C. glabrata*.

## Conclusion

Aldo-keto-reductases are important in intermediary metabolism, detoxification, and stress conditions. The *AKR* gene was highly expressed in azole resistant *C. glabrata*, and may be associated with the biological networks of drug resistance factors of *C. glabrata*. To the best of our knowledge, this study is the first to report the implication of AKR in azole resistance among *C. glabrata* clinical isolates, using the cDNA-AFLP technique. Further investigations are needed to clarify the role of this gene.

## Abbreviations

ABC: ATP binding cassette; AKR: Aldo-keto-reductase; cDNA-AFLP: Complementary DNA-amplified fragment length polymorphism; *CgCDR1*: *Candida glabrata Candida* drug resistance 1; *CgCDR2*: *Candida glabrata Candida* drug resistance 2; *CgPDR1*: *Candida glabrata* pleiotropic drug resistance; *CgSNQ2*: *Candida glabrata* sensitivity to 4-nitroquinoline N-oxide; MFS: Major facilitator superfamily; TDFs: Transcript-derived fragments.

## Competing interests

The authors declare they have no competing interests.

## Authors’ contributions

FZ, MH, and SF contributed to concept and study design, analysis of data, and supervision of sections of the study. SF carried out experimentation and was responsible for the molecular studies, sequence alignment, and analysis of the data. RR assisted with molecular genetics. MS helped in susceptibility testing of antifungal drugs. PK and SR provided scientific advice. SF prepared the manuscript which FZ and MH critically revised. All authors read and approved the final manuscript.
